# Cancer Stem Cells in Oral Cavity Squamous Cell Carcinoma: A Review

**DOI:** 10.3389/fonc.2017.00112

**Published:** 2017-06-02

**Authors:** Ranui Baillie, Swee T. Tan, Tinte Itinteang

**Affiliations:** ^1^Gillies McIndoe Research Institute, Wellington, New Zealand; ^2^Wellington Regional Plastic, Maxillofacial and Burns Unit, Hutt Hospital, Wellington, New Zealand

**Keywords:** oral cavity, squamous cell carcinoma, cancer, cancer stem cells, head and neck

## Abstract

Cancer stem cells (CSCs) have been identified in oral cavity squamous cell carcinoma (OCSCC). CSCs possess the ability for perpetual self-renewal and proliferation, producing downstream progenitor cells and cancer cells that drive tumor growth. Studies of many cancer types including OCSCC have identified CSCs using specific markers, but it is still unclear as to where in the stem cell hierarchy these markers fall. This is compounded further by the presence of multiple CSC subtypes within OCSCC, making investigation reliant on the use of multiple markers. This review examines the current knowledge in CSC markers OCT4, SOX2, NANOG, ALDH1, phosphorylated STAT3, CD44, CD24, CD133, and Musashi-1, specifically focusing on their use and validity in OCSCC CSC research and how they may be organized into the CSC hierarchy. OCSCC CSCs also express components of the renin–angiotensin system (RAS), which suggests CSCs may be novel therapeutic targets by modulation of the RAS using existing medications.

## Introduction

**Figure 1:**
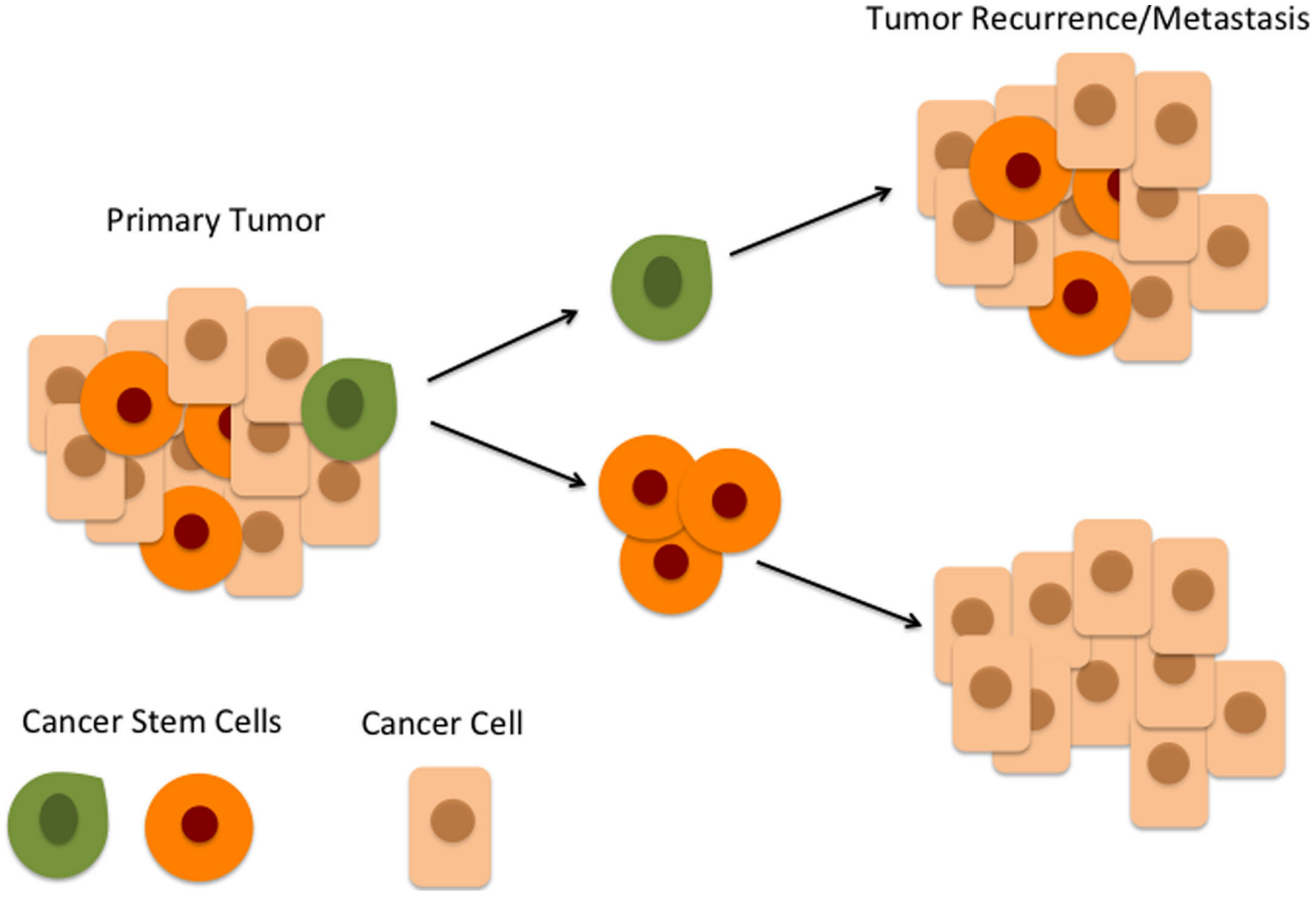
**According to the hierarchical model of cancer, an oral cavity squamous cell carcinoma consists of a heterogeneous population of cells**. At the top of the hierarchy is a small number of cancer stem cells (CSCs) within the peritumoral stroma (green) which differentiate and give rise to downstream CSCs (orange) which in turn give rise to cancer cells (beige). CSCs at the top of the hierarchy (green) are highly tumorigenic and are responsible for tumor recurrence and metastasis.

The overall 5-year survival of oral cavity squamous cell carcinoma (OCSCC) has remained at 50%, largely unchanged for 40 years (1), despite intensive research. This high mortality has been largely attributed to high rates of loco-regional recurrence (2, 3). An emerging hierarchical concept of carcinogenesis proposes that cancer stem cells (CSCs) sit atop a hierarchy of a heterogeneous population of cells within cancer and are defined functionally as a subset of cells that display stemness characteristics, including the ability to asymmetrically divide, resulting in self-renewal of CSCs and the production of heterogeneous populations of cancer cells that are further down the hierarchical ladder (4, 5). CSCs are highly tumorigenic compared to the other cancer cells and are believed to be largely responsible for the biological characteristics of cancer, namely, rapid growth, invasion, and metastasis (Figure 1). CSCs also show a greater capacity for migration, invasion, and proliferation *in vitro* (6, 7), and they generate far larger tumors in immunocompromised xenograft mice with fewer transplanted cells compared to large numbers of unsorted cancer cells (8, 9).

Surgery with adjuvant radiotherapy (RT) and occasionally chemotherapy (ChT) is the mainstay treatment for OCSCC, which effectively reduce total tumor size (10). However, CSCs are predominantly in the inactive G0 phase and thus avoid destruction by RT and ChT that target active cells (11). CSCs in OSCCC are resistant to both RT and ChT agents such as cisplatin (6, 8, 12, 13), carboplatin (6), doxetaxel (6), paclitaxel (6, 14), etoposide (15), gemcitabine (9), and 5-fluorouracil (6–8). Thus, treatment results in an enriching effect on CSCs within the post-treatment cancer cell population (16), providing a plausible rationale for loco-regional recurrence and distant metastasis from RT- and ChT-resistant cells, despite aggressive treatment.

The rapidly accumulating evidence supporting the existence and the role of CSCs in carcinogenesis in recent years is largely due to the advances in cell biology and the discovery of reliable markers of CSC (17). The expression profiles of a number of protein markers have been studied as putative CSC markers within OCSCC tumor samples and cell lines. No single marker has been shown to unequivocally identify CSCs, and it is likely that CSCs exist in an overlapping hierarchy of cell population subsets. Consequently, the majority of CSC characterization research relies on using combinations of these markers. This article reviews the common markers that have been used in CSC research into OCSCC and attempts to place them within the context of a hierarchical model of cancer.

## Embryonic Stem Cell (ESC) Marker Master Regulators

Cancer stem cells in OCSCC express many of the same proteins involved in the core network that regulates ESCs. OCT4 and NANOG are two of the possible six major factors involved in reprogramming of somatic cells into ESC-like states (18, 19). OCT4, NANOG, and SOX2 are considered master regulators for self-renewal and maintenance of the stem cell population in the undifferentiated state (17, 20). Immunohistochemical staining for OCT4, SOX2, and NANOG in OCSCC demonstrates that OCT4 and SOX2 are expressed significantly higher in tumor-adjacent tissue compared to both normal tissue and the tumor (21). However, NANOG is highly expressed in both tumor tissue and peritumoral tissue, compared to normal tissue (21).

### OCT4

The transcription factor OCT4 is a regulator of the POU domain and is critical in early embryogenesis and maintenance of ESC pluripotency (22). As such, OCT4 is commonly used as a marker of “stemness” of primitive cells. OCT4 has also been linked to oncogenic processes (17). It has been suggested that OCT4 plays a role in tumor transformation, tumorigenicity, invasion, and metastasis within OCSCC (23). It has also been proposed that OCT4 promotes tumor initiation by playing a role in the regulation of epithelial–mesenchymal transition (EMT) (13). Expression of OCT4 has been used to define the CSC population in OCSCC in conjunction with other primitive and CSC markers (24–26) and has been shown to contribute to tumorigenicity in murine models (27).

OCT4 has been hypothesized to play an important role in aberrant cell reprogramming resulting in carcinogenesis (28). In moderately differentiated buccal mucosal SCC (BMSCC), expression of OCT4 has been demonstrated in a distinct subpopulation of CSCs within the tumor nests, the peritumoral stroma, and the microvessels within the peritumoral stroma (29). Interestingly in moderately differentiated oral tongue SCC (OTSCC), the expression of OCT4 is restricted to a subpopulation of CSCs within the peritumoral stroma (30). Intriguingly, high levels of expression of OCT4 in OCSCC have been associated with early stage of disease, and better prognosis, and a molecular mechanism explaining this association has yet to be elucidated (21).

### SOX2

The SOX2 protein is a high-mobility SRY-related HMG box transcription factor (31, 32). SOX2 is involved in multiple signal transduction pathways and has been shown to be involved in normal developmental and many pathological processes including cell proliferation, migration, invasion, stemness, tumorigenesis, anti-apoptosis, and chemoresistance (31, 33). SOX2 is known to complex with OCT4 (34), and in murine cell lines has been shown to control downstream embryonic genes including NANOG (20, 35). SOX2 overexpression has been used in combination with other markers, including ALDH1, CD44, OCT4, and NANOG, to identify the CSC population in SCC including OTSCC (26, 30, 31, 36). In BMSCC, SOX2 is expressed within the tumor nests, the peritumoral stroma and the endothelium of the microvessels within the peritumoral stroma (29). In OCSCC and oropharyngeal SCC cell lines, SOX2 is overexpressed in CSCs compared to the parental cell population (37). In OTSCC, SOX2 is expressed by cells that also express SALL4, NANOG, phosphorylated STAT3 (pSTAT3), and CD44 (30). In OCSCC, SOX2 expression is significantly higher in tumor tissue compared to normal tissue and is weakly correlated with OCT4 (21). In addition, SOX2 expression is correlated with small tumor size and early tumor stage, and better disease-free survival (21). SOX2 staining in OCSCC has been demonstrated in both a peripheral and diffuse staining pattern, and the diffuse staining pattern was significantly associated with lymph node metastasis (38). Chien et al. (39) demonstrate that regulation by the Lin28B-Let7 pathway, with the Lin28B^high^-Let7^low^ expression pattern highly correlated with high levels of expression of OCT4 and SOX2 in OCSCC specimens, and a high percentage of CD44^+^/ALDH1^+^ CSC in OCSCC. Overexpression of SOX2 has been demonstrated to enhance invasiveness, anchorage-independent growth, and xenotransplantation tumorigenicity in OCSCC cells. Conversely, silencing SOX2 effectively suppresses the expression of drug resistance and anti-apoptotic genes and increased the sensitivity of the cells to radiation combined cisplatin treatment (33).

### NANOG

NANOG is a homeodomain transcription factor widely known as a marker for primitiveness or “stemness” (20). In murine cell lines, NANOG has been shown to be involved in functionally blocking differentiation and thus maintenance of ESC pluripotency (20, 35). NANOG has been shown to be upregulated in different types of cancers and plays a role in tumor transformation, tumorigenicity, and metastasis within OCSCC (23). Overexpression of NANOG is also correlated with poor differentiation status and chemoresistance (40). In OCSCC, increased expression of NANOG has been found to be associated with poor prognosis (41). NANOG expression has also been confirmed in OTSCC (30). In BMSCC, NANOG is expressed in cells within the tumor nests and the peritumoral stroma (29). In OCSCC and oropharyngeal SCC cell lines, NANOG is overexpressed in the CSC population compared to the parental population (37). Similarly in lip SCC, NANOG is expressed by three distinct putative CSC subpopulations, both within the tumor nests and the peritumoral stroma (42).

### Signal Transducer and Activator of Transcription 3 (STAT3)

Signal transducer and activator of transcription 3 has long been recognized as an oncogene playing a key role in control of cell-cycle progression and anti-apoptosis (43). pSTAT3 plays a critical role in pluripotent stem cells, promoting cell proliferation and resistance to apoptosis, angiogenesis, invasion, and migration (44). In OCSCC, expression of STAT3 within a cell population is localized to the tumor nests that also express CD44, NANOG, and SOX2 (30). Constitutive activation of the STAT3 signaling pathway possesses confirmed oncogenic potential in OCSCC (45). Cross talk with other molecular pathways contributes to STAT3 regulation in cancer (45), and STAT3 is also aberrantly activated by the oversupply of growth factors from the tumor microenvironment (43). For example, Erk1/2 appears to promote serine-pSTAT3, but inhibit tyrosine-pSTAT3 resulting in an overall increased cell growth and varying roles for the different STAT3 phosphorylation sites in OCSCC (45). STAT3 has also been recently found to function co-operatively with SOX2 in the initiation of SCC (32). This further highlights the crucial role of those transcription factors in stem and/or cellular proliferation (44).

Signal transducer and activator of transcription has a dual role in tumor inflammation and immunity by promoting pro-oncogenic inflammatory pathways, including NF-κB and IL-6–GP130–JAK pathways, and by opposing STAT1- and NF-κB-mediated T(h)1 antitumor immune response (46). Continuous deregulation of these genes in tumor cells and the tumor microenvironment by persistently activated STAT3 and NF-κB, in contrast to their tightly controlled regulation in normal physiology, is considered crucial for inflammation and malignant progression (46). In a murine SCC model, forced constitutive activation of pSTAT3 shortens the latency period and increases the number of skin lesions progressing rapidly to SCC (47–54).

## OCSCC Cancer Stem Markers

### CD44

CD44 is a large cell surface hyaluronan receptor protein (36) involved with contrasting roles in both cell migration and adhesion (55). In OCSCC cell lines, CD44 is expressed significantly more highly in CSCs compared to parental cells (37). It has been widely used as a CSC marker in epithelial cancers including OCSCC. CD44 has been identified within normal oral epithelium, carcinoma *in situ*, and in some infiltrating lymphocytes, with the highest expression in carcinoma cells (56, 57). In OCSCC cell lines, Song et al. (15) have demonstrated increased expression of CD44 in side populations that also highly express ABC transporter proteins and Hoechst 33342 efflux, compared to the non-side population.

Overexpression of CD44 within OCSCC has been associated with decreased overall survival (58, 59), increased loco-regional recurrence, and increased resistance to RT, thus exhibiting many of the characteristics of CSCs. In one study of OCSCC, irregular staining of CD44 in tumor cells is shown to be associated with poor tumor differentiation and advanced stage (60). Conversely, another study finds no prognostic significance of CD44v6 expression in OTSCC (61). These differences may, in part, be explained by the expression of CD44v6 by CSCs, as well as inflammatory cells (62). Expression of the variant isoform CD44v6 has also been found to be significantly associated with regional nodal metastasis, pattern of invasion, depth of invasion, perineural invasion, and local recurrence in multiple solid tumors including OCSCC (63). OCSCC cell clones expressing stable levels of CD44 after transfection with CD44 expression vector increases proliferation and migration, inhibition of apoptosis, and cisplatin resistance resulting in a more aggressive tumor phenotype *in vivo* (64).

In addition, cleavage of CD44 regulated by ADAM17 has been found to be necessary for tumor sphere formation in OTSCC cells (65).

Tumors generated from CSC sorted OTSCC cell line (SCC9-CD44^high^) cells demonstrate increased tumorigenicity and increased expression of CK19, B-catenin, E-cadherin, and CD44 when compared with wild-type SCC9 cells. These same tumors show lower expression of CK19/4/15/13, and interestingly low levels of NANOG, Bmi-1, Snail, and Slug (66). However, the role of CD44 as a marker of CSCs is controversial, with many authors arguing that it is actually expressed by more differentiated cells (67). Lee et al. (41) find that increased CD44 expression has limited correlation with high histological grade and late clinical stage. However, Kokko et al. (68) demonstrate no association between expression of CD44 and poor prognosis in OCSCC. A recent study suggests that CD44 loses its expression during induced cellular reprogramming to the undifferentiated state and is actually a marker of partially differentiated cells (69). This may indicate a progressive gain of CD44 expression as CSCs progress to a more differentiated phenotype, and this implies that CD44 is in fact a relatively mature marker, likely downstream of the true CSC population. Interestingly, downregulation of CD44 also leads to reduced expression of OCT4, suggesting that CD44 has a functional role in maintaining stem cell properties (70). CD44^+^/CD133^+^ cells demonstrate higher clonogenic capacity than CD133^−^ cells *in vitro*, while higher CD44 expression is demonstrated in nodal metastases, suggesting a role for CD44 in tumor progression (71).

### CD24

CD24 is a small cell surface glycoprotein involved in cell adhesion and metastasis and has been identified in wide variety of cancer cells (72). A recent study using sorted OCSCC cells in a NOD/SCID murine model suggests that CD24^+^ cells may have angiogenic potential. Tumors generated from CD24^+^ cells isolated show a significantly higher functional capillary density, confirmed by the expression of CD31, than those seeded with CD24^−^ cells (73).

CD44^high^/CD24^low^ cells demonstrate CSC and EMT characteristics, and are able to give rise to all other tumor cell types upon differentiation (74). In OCSCC cell lines, CD44v3^+^/CD24^−^ population demonstrated higher sphere forming capacity, higher drug resistance, and expressed higher mRNA levels of CSC-related genes. Expression of CD44v3 is found to be higher in lymph node metastases and in the invasive portion of tumors and is associated with poorer overall survival (75).

### CD133

CD133 is a pentaspan transmembrane protein that plays a key role in the organization of plasma membrane topology (76, 77). Overexpression of CD133, first identified in hematopoietic stem cells and endothelial progenitor cells (57), is often used as a CSC marker in many solid tumors including OCSCC (23). There remain controversies surrounding the role of CD133 in tumorigenesis with reports regarding the utility of this protein as a CSC marker often being contradictory (77). These conflicting reports are based on the observation that both the CD133^+^ and CD133^−^ cell fractions display similar stemness and differentiation capabilities, and that the CD133^−^ population is in fact more tumorigenic (77).

However, CD133^+^ oral leukoplakia has been shown to be more than three times as likely to progress to OCSCC than CD133^−^ lesions (78). Of all CSC phenotypes studied, OCSCC lesions displaying triple-positive expression of OCT4, NANOG, and CD133, are associated with the worst survival (23). CD133^+^ cells have also been found to co-express CD44, and the CD133^+^/CD44^+^ immunophenotype has been found to correlate significantly with poorer overall survival, supporting the idea that cells expressing these proteins have a more aggressive phenotype (58). The expression of CD133 in oral epithelium increases from normal epithelium, through dysplasia, to carcinoma (79). Pozzi et al. (37) demonstrate that along with multiple CSC and ESC markers, CD133 is more highly expressed in the CSC population compared to the parental normal population. In several cell lines, CD133^+^ cells have been found to overexpress ESC markers, including OCT4 and NANOG, and also display CSC characteristics such as tumor sphere formation, tumorigenicity and chemoresistance (14). In a head and neck SCC cell line, inhibition of CD133 expression significantly reduces proliferation, expression of ESC marker OCT4, but increases the expression of the epithelial differentiation marker CK18, suggesting its role in the maintenance of the CSC-phenotype (80, 81).

### Musashi-1

Musashi-1 is a translational regulator that has been identified within OSCC (17). Musashi-1 expression has been associated with higher stage and poorly differentiated status of OCSCC, and is significantly correlated with CD133, suggesting a functional role for these two proteins in oral carcinogenesis (79).

### ALDH1

Aldehyde dehydrogenase (ALDH) is a cytosolic enzyme responsible for catalyzing the pyridine nucleotide-dependent oxidation of aldehydes to carboxylic acids (82). ALDH has increasingly been used as a CSC marker in OCSCC, with ALDH^+^ cells demonstrating plasticity with the ability to form tumor spheres in serum-free media as well as having the ability to generate ALDH^−^ cells *in vitro* (83). Although there are many isoforms of ALDH, ALDH1 appears to be of particular importance (84). ALDH1 is likely to play a role in malignant transformation of oral leukoplakia to OCSCC given that ALDH1^+^ leukoplakia is more than three times more likely to develop OCSCC (78). Overexpression of ALDH1 is also found to be correlated with nodal metastasis (38). A suppression subtractive hybridization assay shows that the ALDH^+^ subpopulation expresses many known CSC-related genes not seen in the ALDH^−^ population (83). Furthermore, in HNSCC, ALDH^high^ cells are seen to be more tumorigenic than ALDH^low^ cells when implanted into a NOD/SCID murine model (85). In one study of OCSCC, ALDH1^+^ cells display radioresistance and co-expressed Snail, providing evidence of EMT. Interestingly, knockdown of Snail significantly decreased ALDH1 expression and inhibited CSC properties, with resultant decreased tumorigenicity (86).

## Renin–Angiotensin System (RAS)

Cancer stem cells within OCSCC have been found to express components of the RAS. (Pro)renin receptor (PRR), angiotensin II receptor 1 (ATIIR1), and angiotensin II receptor 2 (ATIIR2) are expressed by two CSC subpopulations within OTSCC: one within the tumor nests that express SALL4 and another within the peritumoral stroma that express OCT4 (87). PRR, ATIIR1, and ATIIR2 are localized to the CSC subpopulations within the tumor nests and the peritumoral stroma, while PRR and ACE are localized to the endothelium of the microvessels within the peritumoral stroma (88). These findings suggest CSC as a potential novel therapeutic target by modulating the RAS using commonly used medications such as the aliskiren, a direct renin blocker; β-blockers which reduce renin levels; ACE inhibitors which inhibit conversion of angiotensin I to angiotensin II; and angiotensin receptor blockers which prevent binding of angiotensin II to ATIIR1 and ATIIR2 (89).

## Discussion

**Figure 2:**
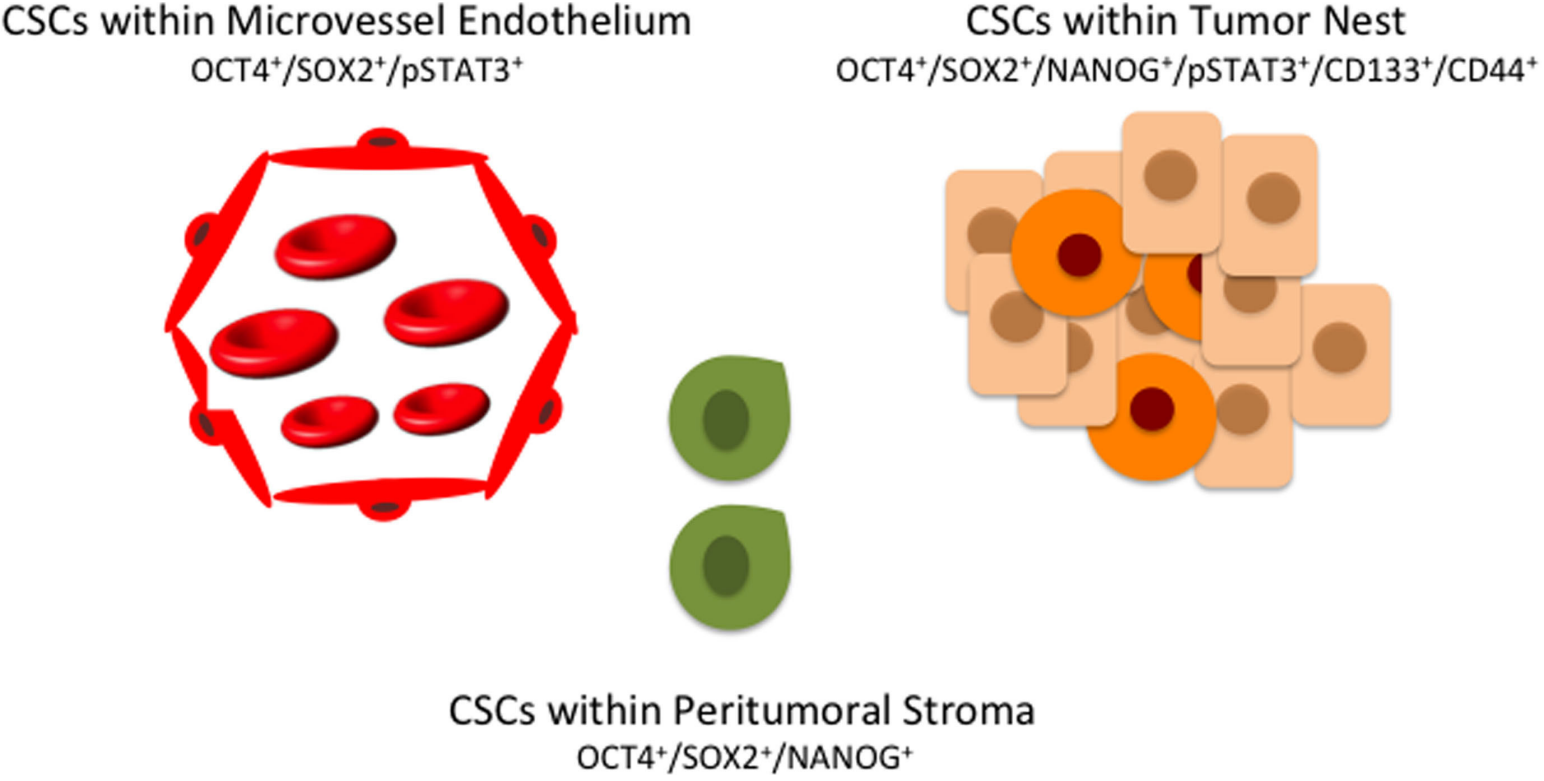
**A hierarchy of cancer stem cells (CSCs) in oral cavity squamous cell carcinoma exists with multiple distinct subpopulations, each expressing overlapping markers**. CSCs within the peritumoral stroma (green) co-express OCT4, SOX4, and NANOG. CSCs within the tumor nests (orange) co-express OCT, SOX2, NANOG, phosphorylated STAT3 (pSTAT3), CD133, and CD44. A further subpopulation of CSCs (red) co-expressing OCT4, SOX2, and pSTAT3 are present on the endothelium of the microvessels within the peritumoral stroma.

The origin of CSCs remains unclear, and many hypotheses have been advanced (90). One of the most accepted theories proposes that CSCs arise as a result of epigenetic or genetic alterations to these resident tissue stem cells (55, 91–93). The CSC concept of cancer is evolving as evidenced from increasingly sophisticated research accumulates (94). Rather than a single small population of CSCs and a large majority of bulk tumor cells, the presence of a complex hierarchy of distinct, genetically heterogeneous subpopulations of CSCs, each expressing an overlapping array of markers (Table 1) is appreciated (94) (Figure 2).

**Table 1:** **Markers for cancer stem cells (CSCs) in oral cavity squamous cell carcinoma (OCSCC)**.

Markers	Roles
OCT4	– Aberrant cell reprogramming resulting in carcinogenesis (28). – Tumor transformation, tumorigenicity, invasion, and metastasis (23, 27). – Role in the regulation of epithelial–mesenchymal transition (EMT) (13). – Conflictingly, high levels of expression also associated with early stage of disease, and better prognosis (21).
NANOG	– Overexpressed in the CSC population compared to the parental population (37). – Associated with tumor transformation, tumorigenicity, and metastasis (23). – Correlated with poor differentiation status and chemoresistance (40). – Increased expression associated with poor prognosis (41).
SOX2	– SOX2 overexpression has been used in combination with other markers to identify the CSC population (26, 30, 31, 36). – Known to complex with OCT4 (34) and control downstream embryonic genes including NANOG (20, 35). – Involved in many pathological processes including cell proliferation, migration, invasion, stemness, tumorigenesis, anti-apoptosis, and chemoresistance (31, 33). – Overexpression of SOX2 has been demonstrated to enhance invasiveness, anchorage-independent growth, and xenotransplantation tumorigenicity in OCSCC cells. – In OCSCC, SOX2 expression is significantly higher in tumor tissue compared to normal tissue and is weakly correlated with OCT4 (21). – Correlated with small tumor size and early tumor stage, and better disease-free survival (21). – Silencing SOX2 effectively suppresses drug resistance and expression of anti-apoptotic genes and increased radiation sensitivity (33).
STAT3	– Well-known oncogene with a role in control of cell-cycle progression and anti-apoptosis (43). – Expression is localized to the tumor nests that also express CD44, NANOG, and SOX2 (30). – Constitutive activation of the STAT3 signaling pathway possesses confirmed oncogenic potential (45). – Cross talk with other molecular pathways contributes to STAT3 regulation in cancer (45). – Aberrantly activated by the oversupply of growth factors from the tumor microenvironment (43, 45). – Function co-operatively with SOX2 in the initiation of SCC (32). – Dual role in tumor inflammation and immunity by promoting pro-oncogenic inflammatory pathways, including NF-κB and IL-6–GP130–JAK pathways, and by opposing STAT1- and NF-κB-mediated T(h)1 anti-tumor immune response (46). – Forced constitutive activation of phosphorylated STAT3 shortens the latency period, and increases the number of skin lesions progressing rapidly to SCC (47–54).
CD44	– Expressed significantly more highly in CSCs compared to parental cells (37). – Widely used as a CSC marker. – Its role as a marker of CSCs is controversial. It may actually be expressed by more differentiated cells (67). – Increased expression has limited correlation with high histological grade and late clinical stage (41), or prognosis (68). – Increased expression of CD44 in side populations that also highly express ABC transporter proteins and Hoechst 33342 efflux (30). – Overexpression is associated with decreased overall survival, increased loco-regional recurrence, and increased resistance to radiotherapy (58, 59). – Associated with poor tumor differentiation and advanced stage (60). – No prognostic significance of CD44v6 expression in oral tongue SCC (61). – Variant isoform CD44v6 associated with regional nodal metastasis, pattern of invasion, depth of invasion, perineural invasion, and local recurrence (63). – Forced stable expression increases proliferation and migration, inhibition of apoptosis, and cisplatin resistance resulting in a more aggressive tumor phenotype *in vivo* (64). – Higher CD44 expression is demonstrated in nodal metastases (71). – Loses expression during induced cellular reprogramming to the undifferentiated state (69), implies that CD44 is in fact a relatively mature marker, likely downstream of the true CSC population. – Downregulation also leads to reduced expression of OCT4, suggesting that CD44 has a functional role in maintaining stem cell properties (70).
CD24	– May have angiogenic potential (73). – CD44^high^/CD24^low^ cells demonstrate CSC and EMT characteristics, and are able to give rise to all other tumor cell types upon differentiation (74). – CD44v3^+^/CD24^−^ cells population demonstrated higher sphere forming capacity, higher drug resistance, and expressed higher mRNA levels of CSC-related genes.
CD133	– Expression of CD133 in oral epithelium increases from normal epithelium, through dysplasia, to carcinoma (79). – Overexpression of CD133 is often used as a CSC marker (23), as C133^+^ cells display CSC characteristics such as tumor sphere formation, tumorigenicity, and chemoresistance (14). – CD133^+^/CD44^+^ cells correlate significantly with poorer overall survival (58). – Inhibition expression significantly reduces proliferation, expression of embryonic stem cell marker OCT4, but increases the expression of the epithelial differentiation marker CK18, suggesting a role in the maintenance of the CSC phenotype (81).
Musashi-1	– Associated with higher stage and poorly differentiated status of OCSCC, and is significantly correlated with CD133, suggesting a functional role (77).
ALDH1	– ALDH1 isoform appears to be of particular importance in OCSCC (84). – ALDH1^+^ leukoplakia was more than 3 times more likely to develop OCSCC (78). – ALDH^+^ cells are able to form tumor spheres in serum-free media and generate ALDH^−^ cells *in vitro* (83). – Overexpression of ALDH1 correlated with nodal metastasis (38). – ALDH^+^ subpopulation expresses many known CSC-related genes not seen in the ALDH^−^ population (83). – ALDH^high^ cells are seen to be more tumorigenic than ALDH^low^ cells when implanted into a NOD/SCID murine model (85). – ALDH1^+^ cells displayed radioresistance and co-expressed Snail, providing evidence of EMT, while Snail knockdown decreased ALDH1 expression and inhibited CSC properties (86).
Components of the RAS	– (Pro)renin receptor (PRR), angiotensin II receptor 1, and angiotensin II receptor 2 are localized to the CSC subpopulations within the tumor nests and the peritumoral stroma, while PRR and ACE are localized to the endothelium of the microvessels within the peritumoral stroma (88).

*RAS, renin–angiotensin system*.

Cancer stem cells also display plasticity (95), and the complexity of these subpopulations increases as tumors progress to more advanced stages (94, 96). Increased density of CSCs, identified by high levels of expression of various primitive cell and CSC markers, has also been shown to be associated with poor prognosis, with much research focused on identifying CSC-related markers that have prognostic value (58, 59, 68).

It has also been shown that both moderately and poorly differentiated OSCC cells demonstrate higher expression of NANOG, SOX2, and OCT4 under hypoxic conditions, suggesting that CSCs share some similarities with induced pluripotent stem cells (97). It is increasingly recognized that the tumor microenvironment plays an important role in supporting tumor growth and metastasis, and contributes to tumor heterogeneity (98). Specialized niche CSC micro environments result from factors that stimulate CSC self-renewal, induce angiogenesis, and recruit cells that facilitate invasion and metastasis (95). It appears that CSCs in SCC switch between two distinct phenotypes that are preferentially migratory or proliferative (99). This plasticity presents an obvious challenge to the development of cancer therapeutics.

Nevertheless, CSC research has promising applications, and directly targeting CSCs has become increasingly appealing as it has the potential to be more effective than traditional approaches while having greater potential for organ preservation and reducing both immediate and long-term off-target toxicity. In addition, most stem cell markers currently used to identify CSCs in basic science research are not sufficiently specific and would be poor targets for direct therapy. Investigations into targeting CSCs vary, including targeting CSC markers and pathways, using epigenetic modulators, immunotherapy agents, and increasing CSC sensitivity to ChT and RT (100).

Oral cavity squamous cell carcinoma tumor spheres display higher expression of CSC and metastasis markers and are also more invasive and are resistant to cisplatin/RT. Increased fucosylation activity identified by upregulation of fucosyltransferases (FUT3 and FUT6) and increased expression of fucosylated polysaccharides such as Sialyl Lewis X are associated with invasion and metastasis (101). Conversely, inhibition of fucosylation negatively affected tumor sphere formation and invasiveness of OCSCC CSCs (101).

Expression of TNF receptor-associated factor 1 has been found to be higher in OCSCC than normal oral mucosa and oral epithelial dysplasia, and is associated with reduced overall survival, Moloney murine leukemia virus integration site 1, Lin28 homolog B, and most importantly ALDH1 in OSCC (102).

A single “silver bullet” that targets CSCs and effectively eliminates cancer remains elusive; however, alternative treatment using a combination of existing medications targeting critical steps of the RAS to control the CSCs may form a future approach to cancer treatment.

## Author Contributions

TI and ST formulated the topic of the review. RB conducted the review. All authors were involved in the drafting, and approved the manuscript.

## Conflict of Interest Statement

The authors declare that the research was conducted in the absence of any commercial or financial relationships that could be construed as a potential conflict of interest. RB, ST, and TI are inventors of the PCT patent application for Cancer Diagnosis and Therapy (No. PCT/NZ2015/050108), and ST and TI are inventors of the PCT patent application for Cancer Therapeutic (62/452479).
